# Empagliflozin and renal histological changes in an experimental model of heart failure in male rats

**DOI:** 10.14814/phy2.70980

**Published:** 2026-06-15

**Authors:** Aline Cavalcanti Toledo Wisnivesky, João Guilherme Ferreira Bertacchi, Bruno Caramelli

**Affiliations:** ^1^ Faculdade de Medicina da USP Unidade de Medicina Interdisciplinar em Cardiologia, Instituto do Coração (InCor) São Paulo Brazil; ^2^ Divisão de Anatomia Patológica, Hospital das Clínicas Faculdade de Medicina da USP São Paulo Brazil

**Keywords:** empagliflozin, heart failure, macrophage polarization, renal congestion, SGLT2 inhibition, tubular atrophy

## Abstract

Renal dysfunction in heart failure (HF) involves congestion‐associated injury, tubular damage, and inflammatory remodeling. Although SGLT2 inhibitors improve renal outcomes in HF, associated renal structural changes in nondiabetic HF remain incompletely characterized. Kidney samples from a previously published cohort of nondiabetic male Wistar rats with myocardial infarction–induced HF treated with empagliflozin (10 mg/kg/day) or vehicle for 4 weeks were analyzed histologically and by immunohistochemistry. Congestion‐associated histological changes, tubular atrophy, tubular morphometric changes, CD68^+^ macrophages, CD206^+^ macrophages, and CD3^+^ T lymphocytes were evaluated. HF animals exhibited increased congestion‐associated histological scores and tubular atrophy compared with sham controls. Empagliflozin was associated with lower congestion‐associated scores in HF animals (median 1 [1] vs. 3.5 [3, 4], *p* = 0.0401) and reduced tubular atrophy (median 1 [0–1] vs. 3 [1–3.75], *p* = 0.0150). Empagliflozin did not reduce total CD68^+^ macrophage counts in HF animals, but was associated with increased CD206^+^ macrophage representation (9.0 ± 1.9 vs. 5.5 ± 1.6 cells/field, *p* = 0.0058), reduced CD3^+^ T‐cell infiltration (12.0 ± 3.5 vs. 21.0 ± 8.0 cells/field, *p* = 0.0051), and increased tubular large‐lumen profiles. Empagliflozin was associated with attenuation of congestion‐associated histological changes, reduced tubular atrophy, tubular morphometric remodeling, and altered renal immune cell composition in nondiabetic HF.

## Graphical abstract

In a non‐diabetic rat model of heart failure, empagliflozin preserved renal structure by attenuating renal changes associated with congestion and tubular atrophy, while modulating inflammatory cell infiltration, characterized by increased CD206‐positive macrophages and reduced CD3‐positive T cells.

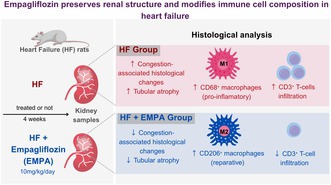

## 1 INTRODUCTION

Heart failure (HF) affects 1%–3% of the global population and is complicated by kidney dysfunction (KD) in nearly half of patients, a condition strongly associated with adverse outcomes (Damman & Testani, 2015; Savarese & Lund, 2017; Ziaeian & Fonarow, 2016). This cardiorenal interaction contributes to higher hospitalization rates, decreased survival, and increased healthcare costs (Damman et al., 2014; Mullens et al., 2020; Ronco et al., 2008; Savarese & Lund, 2017).

Sodium–glucose cotransporter 2 inhibitors (SGLT2i), initially developed as antihyperglycemic agents, are now Class I therapy for HF (Abovich et al., 2023; Heidenreich et al., 2022), reducing cardiovascular death, hospitalizations, and chronic kidney disease (CKD) progression irrespective of diabetes status (McMurray et al., 2019; Packer et al., 2020; Vaduganathan et al., 2022; Zannad et al., 2020). Meta‐analyses confirm approximately 13% reductions in cardiovascular mortality and 20%–27% reductions in the composite of cardiovascular death or HF hospitalization, together with consistent cardiovascular and renoprotective effects (Lauretti et al., 2024; Packer et al., 2020; Vaduganathan et al., 2022; Zannad et al., 2020).

SGLT2 blockade in the proximal tubule promotes glycosuria and natriuresis; however, its benefits extend beyond glucose lowering (DeFronzo et al., 2017; Packer, 2020; Zelniker & Braunwald, 2018). Activation of tubuloglomerular feedback (TGF) has been proposed as a principal mechanism of renal protection (Heerspink et al., 2016; Vallon & Thomson, 2017); however, additional effects, including improved renal oxygen handling, suppression of inflammatory signaling, attenuation of sympathetic activity, and vascular modulation, suggest a broader role for SGLT2 inhibition in tubular protection (Ferrannini et al., 2016; Hattori, 2022; Tonneijck et al., 2017; Verma & McMurray, 2018).

Tubular injury is a major driver of CKD progression, more strongly associated with renal decline than glomerular lesions (Eddy & Neilson, 2018; Schelling, 2016). In HF, renal congestion elevates venous and interstitial pressures, reduces perfusion, induces hypoxia, and activates inflammatory and fibrotic pathways (Afsar et al., 2016; Dupont et al., 2011). Tubular epithelial cells further contribute by releasing cytokines and chemokines that regulate immune responses (Cao et al., 2015). Macrophages are central mediators, polarizing into pro‐inflammatory (M1) or reparative (M2) phenotypes in response to local triggers (Cao et al. 2015; Kimura et al. 2017) Persistent inflammatory activation and lymphocyte recruitment are associated with tubular atrophy and renal senescence, ultimately contributing to progressive loss of renal mass and function (Cao et al., 2015; Kimura et al., 2017; Schelling, 2016). However, the impact of SGLT2 inhibition on congestion‐driven tubular injury and immune remodeling remains insufficiently described in nondiabetic HF.

In the previously published functional study by Borges‐Júnior et al. (2021), using an experimental model of nondiabetic HF rats, empagliflozin was associated with preserved glomerular filtration rate (GFR), improved volume status, and reduced renal apoptosis in the presence of persistent systolic dysfunction. However, the structural renal correlates underlying these functional findings were not investigated, leaving an important gap in the interpretation of the observed renoprotective effects.

The present study aimed to evaluate renal alterations characteristic of heart failure and to determine the effects of empagliflozin on these changes. To this end, we characterized renal histological and immune alterations, focusing on congestion‐associated histological changes, tubular atrophy, and immune cell infiltration, key structural features of renal involvement in HF (Damman & Testani, 2015). Importantly, this analysis was performed using kidney samples derived from the same experimental cohort previously described by Borges‐Júnior et al. (2021), corresponding to the same animals included in the prior functional study.

## 2 MATERIALS AND METHODS

### 2.1 Study design and ethical approval

The kidney samples analyzed in the present study were derived from Borges‐Júnior et al. (2021) study, a previously published experimental cohort investigating the functional cardiorenal effects of empagliflozin in nondiabetic HF rats. Detailed cardiac performance indices for this cohort, including echocardiographic parameters and BNP measurements, as well as renal functional parameters such as urinary glucose and sodium excretion, glomerular filtration rate (GFR), and urinary volume, were previously reported and are referenced here to provide physiological context for the present structural analyses (Borges‐Júnior et al., 2021). The current investigation focused exclusively on additional histological and immunohistochemical analyses not previously reported.

All experiments were conducted in accordance with the ethical guidelines for animal research established by the Brazilian College of Animal Experimentation and approved by the Institutional Animal Care and Use Committee of the University of São Paulo Medical School (protocol No. 1846/2023).

### 2.2 Animal model and experimental groups

The Kidney samples were derived from Male Wistar rats (3–4 months old, 220 ± 20 g) that underwent ligation of the left anterior descending coronary artery to induce myocardial infarction, as previously described (Johns & Olson, 1954) or sham surgery. Coronary ligation–induced myocardial infarction in rats is a well‐established experimental model of chronic systolic dysfunction and post‐infarction HF, with cardiac impairment related to infarct size and progressive neurohumoral activation. This model has also been used to study cardiorenal interactions, including altered renal hemodynamics and sodium excretion after myocardial infarction (Cowie & Fisher, 2020; Layton & Vallon, 2018; Seyer‐Hansen et al., 1980). HF was confirmed 4 weeks after surgery by elevated BNP levels and reduced fractional area change (FAC; the percentage change in LV cross‐sectional area between diastole and systole), both of which differed significantly from the sham groups (*p* < 0.001) (Borges‐Júnior et al., 2021). Animals meeting the predefined HF criteria (BNP >1.0 ng/mL and FAC <40%) were included in the HF groups.

Animals were then randomized to receive empagliflozin (10 mg/kg/day) or vehicle for 4 weeks. Tablets of empagliflozin (Jardiance®, 25 mg; Boehringer Ingelheim Pharma GmbH & Co. KG, Germany) were obtained from a local pharmacy and processed by a specialized compounding facility (Rhoster, Araçoiaba da Serra, São Paulo, Brazil) for homogeneous incorporation into chow and control animals received identical chow without empagliflozin.

Food and water intake were assessed over a 24‐h period using metabolic cages, as previously described (Borges‐Júnior et al., 2021). Rats were individually housed in metabolic cages (Tecniplast, Buguggiate, VA, Italy), and food and water consumption were measured and normalized to body weight. As reported in the parent cohort, empagliflozin‐treated animals consumed the medicated chow and exhibited higher food intake than untreated animals and increased urinary glucose excretion, confirming effective drug intake and SGLT2 inhibition.

Animals were housed under controlled environmental conditions (12‐h light–dark cycle, regulated temperature and humidity) with ad libitum access to food and water. At the end of treatment, animals were anesthetized with ketamine and xylazine, organs were harvested, and euthanasia was performed by cervical dislocation in accordance with institutional guidelines.

### 2.3 Sample size

Kidney samples were obtained from the following groups: Sham (*n* = 7), Sham + EMPA (*n* = 8), HF (*n* = 10), and HF + EMPA (n = 10). Samples with staining artifacts or technical inconsistencies that could interfere with quantitative assessment were excluded before blind analysis. Group sizes for immunohistochemistry and morphometry are indicated below and in the corresponding figure legends.

Because the present work represents a secondary histological analysis of a previously established cohort, no priori sample size calculation was performed specifically for the histological endpoints evaluated here.

### 2.4 Histological analysis

The kidneys were sectioned along the mid‐frontal plane immediately after harvesting, fixed in 10% formalin for 24 h, transferred to 70% ethanol, embedded in paraffin, and cut into 4‐μm sections mounted on silanized slides.

Histological images from immunohistochemistry and Picrosirius Red–stained sections were acquired from one renal section per animal, with 7–10 randomly selected cortical fields analyzed per section at 200× magnification using a light microscope (Leica Microsystems, Wetzlar, Germany) coupled to Quantimet Leica software. All acquired images were subsequently analyzed in a blinded manner using ImageJ software (National Institutes of Health, Bethesda, MD, USA).

### 2.5 Immunohistochemistry

Kidney sections were deparaffinized, rehydrated, and subjected to antigen retrieval in a 10 mM citric acid buffer (pH 6) at high temperature. Sections were washed with Tris‐buffered saline containing 0.1% Tween 20 (TBST). Endogenous peroxidase activity was blocked using 3% H_2_O_2_ for 3 min, repeated five times, followed by washing in TBST. Nonspecific binding was blocked with 2% normal goat serum (005000121, Jackson ImmunoResearch) for 30 min at room temperature.

Sections were incubated overnight at 4°C with antibodies against CD68 (1:400; ab31630, Abcam), CD206 (1:500; ab300621, Abcam), and CD3 (1:400; 559975, BD Biosciences). Secondary detection was performed using Biotin‐SP–conjugated AffiniPure donkey anti‐rabbit IgG (1:500; 711065152, Jackson ImmunoResearch) or anti‐mouse IgG (1:500; 715065150, Jackson ImmunoResearch), followed by incubation with the avidin–biotin–peroxidase complex (ABC Elite kit; Vector Laboratories). Immunoreactivity was visualized with 3,3′‐diaminobenzidine (DAB; Sigma‐Aldrich Chemie), and sections were counterstained with Harris hematoxylin.

### 2.6 Morphometric analysis

#### 2.6.1 Renal congestion‐associated histological changes and tubular atrophy

Renal congestion‐associated histological changes and tubular atrophy were evaluated on three renal sections per animal: one stained with hematoxylin and eosin (H&E), one with Masson's trichrome, and one with periodic acid–methenamine silver (PAMS). Two renal pathologists blinded to the experimental groups simultaneously evaluated the histological slides using a light microscope and assigned semi quantitative scores for each criterion according to the extent of the affected area: score 0 (absence), score 1 (≤1%), score 2 (1%–5%), score 3 (5%–10%), and score 4 (10%–20%). Congestion‐associated histological changes were defined by the presence of vascular and interstitial erythrocyte‐rich areas, reflecting blood accumulation within the renal microcirculation. In the setting of heart failure, increased renal venous pressure is transmitted to the intrarenal venous and capillary networks, leading to engorgement and distension of small vessels, including peritubular capillaries. Accordingly, the congestion‐related score represents a structural surrogate of renal venous congestion rather than a direct hemodynamic measurement (Afsar et al., 2016; Dupont et al., 2011; Husain‐Syed et al., 2021). Tubular atrophy was defined by thickening and wrinkling of the tubular basement membrane accompanied by reduced tubular epithelial cell height, consistent with established histopathological descriptions of tubular injury (Eddy & Neilson, 2018; Schelling, 2016).

#### 2.6.2 Tubular morphometric changes

Tubular morphometric changes were assessed on Picrosirius Red–stained sections (*n* = 6 per group). Tubular profiles exhibiting a lumen‐to‐tubular tissue area ratio >1 on histological cross‐sections were quantified as meeting the predefined morphometric criterion as an indirect indicator of possible enlargement of tubules after SGLT2 inhibitors treatment, as demonstrated previously in an experimental study with healthy animals (Sinha et al., 2023). Only tubules with a near‐transverse orientation and well‐defined boundaries were included in the analysis (Sinha et al., 2023).

### 2.7 Statistical analysis

Continuous quantitative variables were analyzed using two‐way ANOVA followed by Tukey's post hoc test, except for tubular morphometric data, for which prespecified pairwise comparisons between treated and untreated groups were performed using Welch's *t*‐test due to unequal variance between groups. Data are presented as mean ± standard deviation (SD). Semiquantitative histological scores (congestion‐associated renal changes and tubular atrophy), derived from ordinal scales, were assessed using the Kruskal–Wallis test followed by Mann–Whitney *U* test and presented as median and interquartile range. A two‐tailed *p* value <0.05 was considered statistically significant. Statistical analyses were performed using GraphPad Prism version 10 (GraphPad Software, San Diego, CA, USA).

## 3 RESULTS

### 3.1 Functional context

As previously reported by Borges‐Júnior et al. (2021), the HF animals exhibited significant systolic dysfunction compared with sham controls. Empagliflozin was associated with modest improvement in fractional area change, and the HF phenotype remained evident at the time of renal tissue collection. In the presence of persistent systolic impairment, empagliflozin preserved GFR and improved volume status in HF animals. Urinary sodium and glucose excretion were significantly increased in treated groups, confirming effective SGLT2 inhibition. These previously published functional findings provide physiological context for interpretation of the renal structural observations reported here.

### 3.2 Congestion‐associated histological changes, tubular atrophy and tubular morphometric changes

**FIGURE 1:**
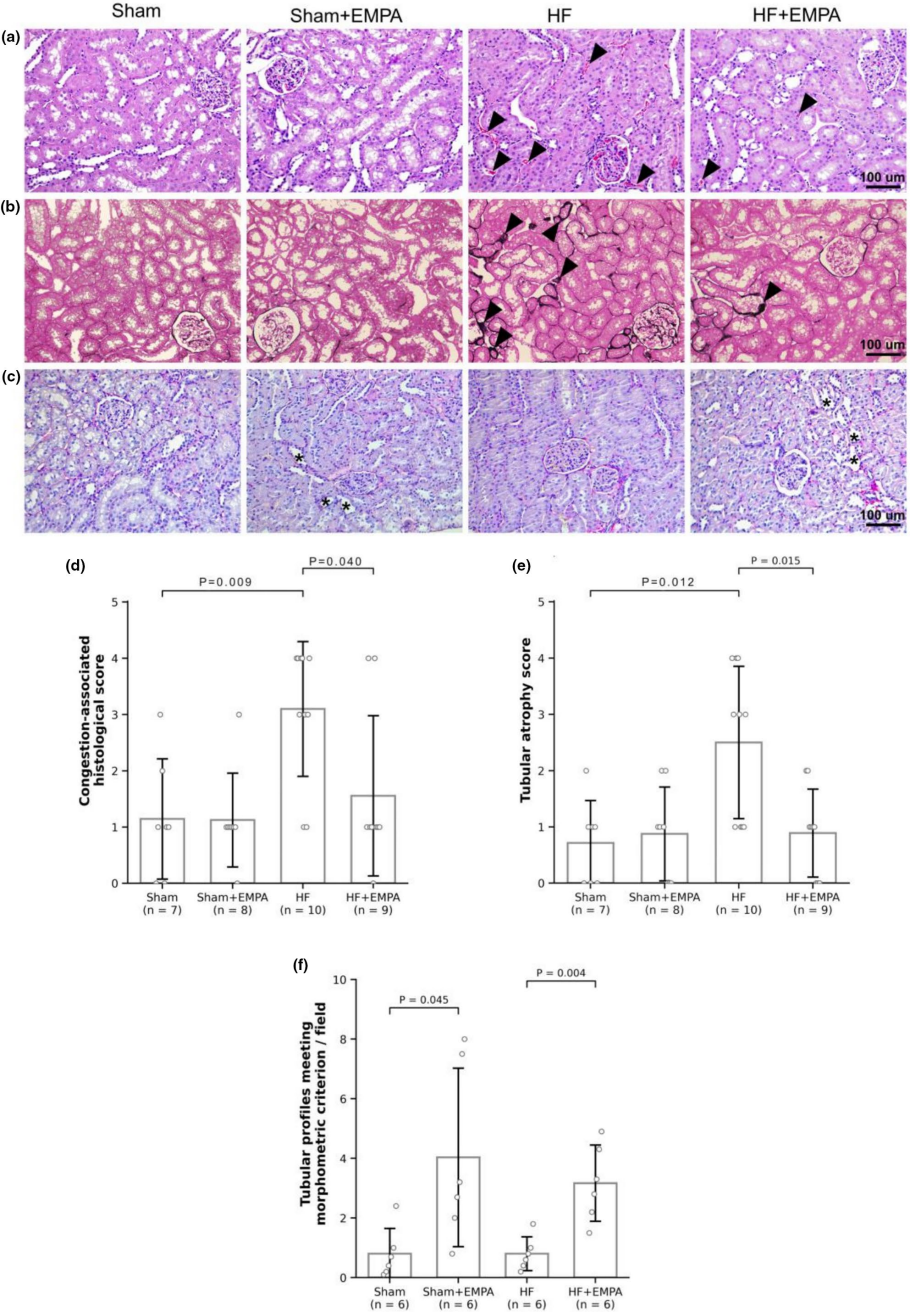
Renal structural alterations in experimental heart failure and effects of empagliflozin. (a–c) Representative histological sections acquired at 200× magnification in Sham, Sham+EMPA, HF, and HF + EMPA groups. (a) Congestion‐associated renal histological changes on H&E staining; arrowheads indicate erythrocyte‐rich vascular/interstitial areas. (b) Tubular atrophy on PAMS staining; arrowheads indicate thickened and wrinkled tubular basement membranes.(c) Tubular profiles assessed by Picrosirius Red staining; asterisks indicate tubular profiles with larger luminal areas meeting the predefined morphometric criterion. (d) Semiquantitative congestion‐associated histological score. (e) Semiquantitative tubular atrophy score. (f) Quantification of tubular profiles with larger luminal areas per field, defined as profiles exhibiting a lumen‐to‐tubular tissue area ratio >1. Data are shown with individual data points. Semiquantitative scores are presented as median with interquartile range and were analyzed using the Kruskal–Wallis test followed by prespecified Mann–Whitney comparisons. Quantitative morphometric data are presented as mean ± SD and were analyzed using prespecified Welch's *t*‐tests comparing treated groups with their respective untreated controls. *p* values and group sizes are indicated in the figure.

Experimental HF was associated with significant renal structural abnormalities, including increased congestion‐associated histological changes and tubular atrophy compared with sham animals (Figure 1).

Congestion‐associated histological scores were significantly higher in HF rats than in sham controls (median 3.5 [3, 4] vs. 1 [0–2], *p* = 0.009; Figure 1a,d). Empagliflozin treatment was associated with lower congestion‐associated histological scores in HF animals compared with untreated HF rats (median 1 [1] vs. 3.5 [3, 4], *p* = 0.0401).

HF animals showed greater tubular atrophy than sham controls (median 3 [1–3.75] vs. 1 [0–1], *p* = 0.0121; Figure 1b,e), whereas empagliflozin treatment was associated with reduced tubular atrophy compared with untreated HF animals (median 1 [0–1] vs. 3 [1–3.75], *p* = 0.0150).

Empagliflozin treatment was additionally associated with an increased frequency of tubular profiles meeting the predefined morphometric criterion of lumen area (Figure 1e) in Sham+EMPA and HF + EMPA animals compared with their respective untreated controls (4.03 ± 2.99 vs. 0.80 ± 0.85 profiles/field, *p* = 0.0451; and 3.17 ± 1.28 vs. 0.80 ± 0.57 profiles/field, *p* = 0.004, respectively). Because this parameter was operationally defined from histological cross‐sections and kidneys were not vascularly perfused before fixation, morphometric findings involving luminal structures and congestion‐associated changes should be interpreted with caution.

### 3.3 Renal immune cell composition

**FIGURE 2:**
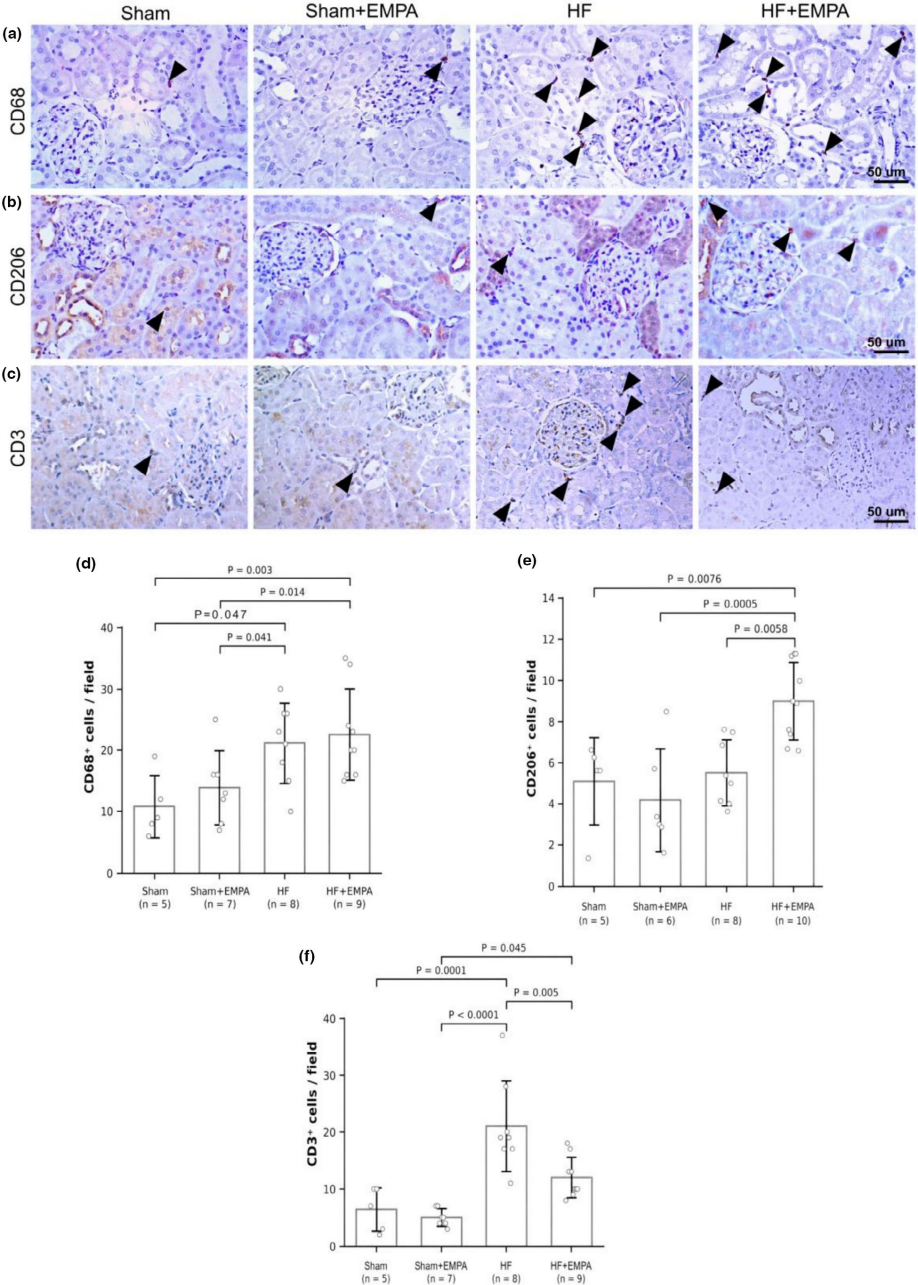
Renal immune cell composition in experimental heart failure and effects of empagliflozin. (a–c) Representative immunohistochemical sections acquired at 200× magnification illustrating CD68^+^ cells, CD206^+^ cells, and CD3^+^ T lymphocytes in Sham, Sham+EMPA, HF, and HF + EMPA groups; arrowheads indicate positively stained cells. (d) Quantification of CD68^+^ cells per field. (e) Quantification of CD206^+^ cells per field. (f) Quantification of CD3^+^ cells per field. Data are shown with individual data points. Quantitative immunohistochemical data are presented as mean ± SD and were analyzed using two‐way ANOVA followed by Tukey's multiple‐comparison test. *p* values and group sizes are indicated in the figure.

Experimental HF was associated with increased renal immune cell accumulation (Figure 2). Quantitative analysis demonstrated higher numbers of CD68^+^ macrophages in HF animals compared with sham controls (21.1 ± 6.6 vs. 10.8 ± 5.1 cells/field, *p* = 0.0047; Figure 2a,d). We did not observe a difference in total macrophage infiltration in HF animals associated with Empagliflozin treatment (22.6 ± 7.5 vs. 21.1 ± 6.6 cells/field, *p* = 0.969).

In contrast, CD206^+^ macrophages were significantly increased in HF animals treated with empagliflozin compared with untreated HF rats (9.0 ± 1.9 vs. 5.5 ± 1.6 cells/field, *p* = 0.0058; Figure 2b,e), suggesting a shift in macrophage composition toward a reparative M2 phenotype. This effect was not observed between sham groups.

CD3^+^ T‐cell infiltration was significantly elevated in HF animals compared with sham controls (21.0 ± 8.0 vs. 6.4 ± 3.8 cells/field, *p* = 0.0001; Figure 2c,f) and was reduced following empagliflozin treatment (12.0 ± 3.5 vs. 21.0 ± 8.0 cells/field, *p* = 0.005).

## 4 DISCUSSION

This study builds on previous functional data by providing a histological evaluation of how empagliflozin alters kidney structure and immune responses in nondiabetic male rats with heart failure (HF). The main findings were that HF was associated with higher congestion‐associated histological scores, greater tubular atrophy, increased CD68^+^ macrophage accumulation, and increased CD3^+^ T‐cell infiltration, whereas empagliflozin treatment was associated with lower congestion‐related histological scores, reduced tubular atrophy, increased CD206^+^ macrophage representation, and reduced CD3^+^ T‐cell infiltration. Importantly, the effects associated with empagliflozin treatment were observed in the presence of persistent systolic dysfunction in the parent cohort.

The present findings represent a secondary histological analysis of a previously published cohort and should be interpreted as associative rather than mechanistic hypothesis. Indeed, this study was not designed to determine whether the observed immune and structural changes were primary or secondary effects of empagliflozin treatment. Therefore, causal inferences have been deliberately limited.

Accordingly, the semiquantitative histological score used in the present study should be interpreted as a condition reflecting congestion‐associated structural changes compatible with renal congestion rather than direct quantification of renal congestion itself. Even with this distinction, the observation that HF animals exhibited more pronounced erythrocyte‐rich vascular and interstitial histological changes than sham animals, and that these changes were attenuated in HF rats treated with empagliflozin, is consistent with the broader concept that decongestion itself may contribute to renal structural preservation in HF.

These findings are also consistent with the recognized role of renal congestion in cardiorenal dysfunction, as increased venous and interstitial pressures impair renal perfusion and promote tubular hypoxia, as well as with previous studies demonstrating that empagliflozin restores euvolemia under normoglycemic conditions through osmotic and natriuretic effects mediated, at least in part, by SGLT2 and NHE3 inhibition (Borges‐Júnior et al., 2021; Cowie & Fisher, 2020; Layton & Vallon, 2018). Specifically, a randomized trial involving predominantly non‐diabetic heart failure (HF) patients showed that empagliflozin lowers systolic blood pressure and decreases markers of both plasma and extracellular volume, indicating a systemic decongestive effect (Jensen et al., 2021).

In this context, attenuation of histological changes compatible with renal congestion may represent an important contributor to tubular preservation (Aart‐van der Beek et al., 2022; Bailey et al., 2022; Boorsma et al., 2022; Upadhyay, 2024) Because the kidney is encapsulated, congestion in heart failure increases interstitial pressure, leading to compression of capillaries and tubules and impairing renal perfusion beyond the effects of reduced cardiac output alone (Aart‐van der Beek et al., 2022; Boorsma et al., 2022).

The association between empagliflozin and lower tubular atrophy is also noteworthy. Tubular injury is increasingly recognized as a major component of renal dysfunction in HF, and previous work in this cohort demonstrated preservation of GFR and improved volume status in the presence of ongoing systolic dysfunction. In addition, SGLT2 inhibitors have been associated with reductions in established markers of tubular injury, including kidney injury molecule‐1 (KIM‐1), across experimental models of congestion and diabetic renal injury, as well as in clinical heart failure populations (Ahmed et al., 2021; Damman et al., 2011; Dekkers et al., 2018; Gojaseni et al., 2024; Nakatsukasa et al., 2022; Packer et al., 2023). Although molecular markers of tubular injury were not assessed in the present study, the histological findings are directionally consistent with the possibility that empagliflozin may help preserve tubular structure in this setting. However, causality cannot be inferred from the present design.

Empagliflozin was not associated with a reduction in total CD68^+^ macrophage counts in HF but was associated with greater CD206^+^ macrophage representation and lower CD3^+^ T‐cell infiltration in HF animals. Importantly, the increase in CD206^+^ cells was observed only in the HF context, not in sham animals. This suggests that the immune association may be context dependent and may reflect the presence of HF‐related renal injury and a modified tissue microenvironment rather than a baseline effect of empagliflozin in healthy renal tissue.

Although CD206 expression is commonly associated with a reparative macrophage phenotype (Cao et al., 2015; Kimura et al., 2017), the present data do not allow definitive conclusions regarding functional macrophage polarization. Experimental evidence from other renal injury models suggests that SGLT2 inhibition may influence macrophage phenotype and inflammatory signaling (Cao et al., 2015; Wang et al., 2021). However, in the current study, CD206^+^ cell abundance should be interpreted as reflecting phenotypic representation rather than confirmed functional polarization. Thus, the increased presence of CD206^+^ cells may indicate a relative shift toward an M2‐like profile that may be modulated by changes within the renal microenvironment.

This interpretation is consistent with a meta‐analysis of experimental studies demonstrating reductions in inflammatory markers—including IL‐6, C‐reactive protein, TNF‐α, and monocyte chemoattractant protein‐1—following SGLT2 inhibitor treatment independent of glycemic status (Theofilis et al., 2022).

Collectively, these observations support a model in which attenuation of congestion‐associated renal changes, structural preservation, and alterations in immune cell composition occur in parallel in non‐diabetic heart failure. Additional cellular pathways have been proposed in other experimental contexts (Bruckert et al., 2022; Huang et al., 2022; Marx & Packer, 2021; Semo et al., 2023), but these mechanisms were not directly evaluated in the present study.

Interestingly, empagliflozin treatment was associated with an increased frequency of tubular profiles with larger luminal areas meeting the predefined morphometric criterion in both sham and HF animals. This finding may be related to tubular morphological changes previously described with SGLT2 inhibition and chronic diuretic exposure, both of which have been associated with tubular hypertrophy‐related adaptations. Although the present finding should not be interpreted as direct evidence of tubular hypertrophy, it may be consistent with adaptive tubular remodeling related to changes in tubular transport workload. SGLT2 inhibition modifies proximal sodium and glucose handling and increases downstream solute and water delivery, in line with the tubular hypothesis of nephron filtration (Vallon & Thomson, 2020). Experimental evidence also shows that increased tubular workload induced by furosemide can promote tubular hypertrophy‐related structural adaptation (Kobayashi et al., 1995). Similarly, empagliflozin increased kidney weight and hypertrophy at specific nephron segments (Sinha et al., 2023). However, because this parameter was operationally defined only from histological cross‐sections and kidneys were not vascularly perfused before fixation, these findings should be interpreted cautiously.

Overall, the present study adds structural information to previously reported functional observations from the same nondiabetic HF animal cohort. Rather than demonstrating a direct mechanism, the data support an association between empagliflozin treatment and renal structural preservation accompanied by altered immune cell composition in HF.

## 5 LIMITATIONS

Several limitations should be acknowledged. First, this study is observational in nature and does not establish causal relationships between congestion‐related histological changes, tubular preservation, and immune remodeling. Second, molecular markers of tubular injury and hypoxia were not directly assessed, limiting mechanistic interpretation. Third, immune characterization was restricted to selected markers and does not define macrophage functional phenotype. Fourth, because kidneys were not vascularly perfused before fixation, luminal and vascular histological features may have been influenced by tissue processing and residual intravascular blood. Fifth, the present study represents a secondary analysis of a previously established cohort, and no a priori power calculation was performed specifically for the histological endpoints evaluated here. Finally, the long‐term implications of tubular hypertrophy were not assessed.

## 6 CONCLUSION

In summary, empagliflozin treatment was associated with lower congestion‐related renal histological scores, reduced tubular atrophy, and altered immune cell composition in a nondiabetic model of heart failure in male rats. These findings extend prior functional observations in this cohort but should be interpreted as associative rather than mechanistic hypotheses.

## AUTHOR CONTRIBUTIONS

**Aline Cavalcanti Toledo Wisnivesky:** Conceptualization; data curation; formal analysis; funding acquisition; investigation; methodology; project administration; resources; software. **Bruno Caramelli:** Conceptualization; data curation; formal analysis; funding acquisition; investigation; methodology; project administration; resources; supervision; validation; visualization. **João Guilherme Ferreira Bertacchi:** Formal analysis; investigation; methodology.

## CONFLICT OF INTEREST STATEMENT

The authors declare no conflicts of interest.

## Data Availability

The datasets generated and analyzed during the current study, including quantitative histological and immunohistochemical measurements, are available from the corresponding author upon reasonable request.
