# Correction to “Empagliflozin and renal histological changes in an experimental model of heart failure in male rats”

**DOI:** 10.14814/phy2.71170

**Published:** 2026-11-10

Wisnivesky, A. C. T. , Bertacchi, J. G. F. , & Caramelli, B. (2026). Empagliflozin and renal histological changes in an experimental model of heart failure in male rats. Physiological Reports, 14, e70980. 10.14814/phy2.70980.PMC1326916842298367

In the published article, the funding acknowledgment for the São Paulo Research Foundation (FAPESP), Brazil, was inadvertently omitted. The following statement should be included in the Acknowledgments section:

“This research was supported by the São Paulo Research Foundation (FAPESP), Brazil (grant number 2020/02668‐2).”

We apologize for this error. The originally published article has also been updated accordingly.

